# Implementation of Machine Learning in Heart Failure Trials

**DOI:** 10.1007/s11897-026-00752-1

**Published:** 2026-03-28

**Authors:** Letizia Rosa Romano, Marta Scimeca Odorico, Antonio Curcio

**Affiliations:** 1 Division of Cardiology, Annunziata Hospital, 87100 Cosenza, Italy; 2 School of Cardiovascular and Metabolic Health, University of Glasgow, Glasgow, G12 8QQ UK; 3 Department of Pharmacy, Health and Nutritional Sciences, University of Calabria, Rende, 87036 Italy

**Keywords:** Heart failure, Machine learning, Clinical trials, Remote monitoring, Risk prediction

## Abstract

**Purpose of Review:**

Heart failure (HF) is a heterogeneous syndrome that challenges the design and interpretation of results from clinical trials. This review examines how machine learning (ML) can address methodological constraints of traditional trial models, such as rigid eligibility criteria, fixed endpoints, and limited external validity.

**Recent Findings:**

By integrating multimodal data from electronic health records, imaging, biomarkers, and wearables, ML enhances patient stratification, refines inclusion criteria, and improves prediction of mortality, HF hospitalization, and treatment response. It also enables adaptive trial designs, continuous monitoring, and dynamic endpoint evaluation. Despite these advances, challenges related to bias, interpretability, and regulatory adaptation persist.

**Summary:**

ML complements rather than replacing conventional methodologies, and promotes more adaptive, inclusive, and patient-centered HF research. Responsible implementation—based on transparency, rigorous validation, and fairness—may redefine evidence generation and bridge clinical trials with real-world practice.

## Introduction

Conventional clinical trials frequently struggle to capture the complexity of real-world patient populations. Strict eligibility criteria often limit representativeness, while standard endpoints may inadequately reflect heterogeneous treatment responses. Moreover, the systematic exclusion of older, multimorbid, or atypical individuals reduces the generalizability of trial findings to routine clinical practice [1]. These limitations are particularly evident in heart failure (HF), a highly heterogeneous syndrome encompassing diverse etiologies, pathophysiological mechanisms, and clinical phenotypes. HF is commonly classified according to left ventricular (LV) ejection fraction (EF) into HF with reduced EF (HFrEF, < 40%), mildly reduced EF (HFmrEF, 40 ÷ 49%), and preserved EF (HFpEF, ≥ 50%), reflecting distinct biological substrates and therapeutic implications [2]. This variability may complicate the design, interpretation, and external validity of randomized clinical trials (RCTs), which nonetheless remain the cornerstone of evidence generation in cardiology [3].

Data-driven approaches, particularly machine learning (ML), are increasingly explored to overcome limitations of conventional HF trials. ML enables analysis of high-dimensional data to identify latent patterns, supporting refined phenotypic stratification, integrated prognostic modeling, and improved prediction of HF hospitalization (HFH) and mortality [4]. By integrating multimodal data, including electronic health records (EHR), electrocardiographic (ECG) data, imaging, biomarkers, and wearable devices (WD), ML may enhance external validity and clinical relevance [5]. However, most applications remain retrospective, with limited translation due to scarce prospective validation and lack of standardized frameworks, highlighting the need for rigorous methodological evaluation [6].

This manuscript was conducted as a narrative, semi-systematic review. PubMed/MEDLINE, Scopus, and Web of Science were searched for studies published between January 2020 and November 2025, using combinations of the following keywords: *heart failure*,* machine learning*,* artificial intelligence*,* clinical trials*,* electronic health records*,* wearables*,* risk prediction*,* and phenotyping*. Eligible articles included original studies, systematic reviews, and consensus documents addressing ML applications in HF trials or trial-related processes.

The aim of this review is to examine how ML may address key limitations of conventional HF trial design, spanning patient stratification, eligibility refinement, prognostic modeling, and outcome prediction, while also considering methodological, regulatory, and ethical challenges. A balanced appraisal of opportunities and constraints is essential to define the conditions under which ML can support more efficient, inclusive, and impactful HF trials.

## Principles of Machine Learning Relevant to Clinical Research

### Complexity of Clinical Trials and Emerging Opportunities for ML

Over the past decade, clinical trial design has evolved toward greater methodological rigor, driven by expanded endpoints, stricter eligibility rules, innovative methodologies, and evolving regulatory requirements. Despite substantial innovation in trial formats, endpoints, and regulatory frameworks, study duration has continued to lengthen, and success rates remain limited. Analysis of more than 16,000 trial protocols demonstrates that modern trials are characterized by a marked rise in procedural and design complexity, largely fueled by the expansion of endpoints and increasingly restrictive inclusion and exclusion criteria [7].

While most ML applications have focused on clinical care, their potential to transform clinical research has only recently gained attention [8]. ML refers to a set of computational techniques that enable algorithms to identify patterns and make predictions by learning from data, without being explicitly programmed to follow predefined rules. Table 1 reports key terminology and its potential applications in HF research.

**Table 1:** Key Terminology and Definitions Relevant to Heart Failure Clinical Trials

Term	Definition	Relevance to HF research & trial methodology	References
Artificial Intelligence	Field that develops systems capable of “intelligent” tasks (perception, prediction, decision-making).	Umbrella for ML/DL used for diagnosis, prognosis, and trial optimization (standards, transparency, clinical adoption).	Indolfi et al. [9], 2025 Danilov & Aronow et al. [10], 2023
Machine Learning	Algorithms that learn patterns from data to predict or classify without explicit rule-based programming.	Outcome prediction (mortality, readmission), EHR/NLP-based screening, phenotypic enrichment, dynamic endpoints.	Waring et al. [11], 2020 Banerjee et al. [12], 2021 Averbuch et al. [8], 2022
Deep Learning	ML subsets using multi-layer neural networks that learn hierarchical, non-linear representations.	Analysis of ECG/imaging, EF estimation, HF classification and prognosis; requires large datasets and external validation.	Mishra & Reddy [13], 2021 Han et al. [14], 2020
Supervised learning	Trained on labelled data (known input→output) to perform classification or regression.	Data-driven prognostic scores for mortality/readmission, treatment-response modeling; supports adaptive/randomized designs.	Waring et al. [11], 2020 Mpanya et al. [15], 2023
Unsupervised learning	Learn without labels to discover clusters/latent structure.	HF/HFpEF phenomapping for biologically coherent enrichment and predefined subgroup design in trials.	Banerjee et al. [12], 2021 Meijs et al. [16], 2023 Jentzer et al. [17], 2024
Reinforcement learning	Agent learns optimal policies via sequential reward/penalty feedback.	Potential for real-time adaptive decisions (dose/timing, adaptive trial strategies); still emerging.	Jayaraman et al. [18], 2024
Electronic Health Record	Longitudinal digital patient chart (clinical and family history, notes, diagnoses, meds, tests, imaging).	Primary source for data extraction/diagnosis, automated pre-screening, outcome adjudication via NLP, EF-trajectory modeling.	Girouard et al. [19], 2024 Vuori et al. [20], 2023 Farajidavar et al. [21], 2022 Adekkanattu et al. [22], 2023
Natural Language Processing	ML/DL methods to extract information from unstructured text.	Rapid eligibility screening, cohort assembly, endpoint adjudication from clinical text in HF RCTs.	Girouard et al. [19], 2024
Multimodal data	Integration of heterogeneous sources (clinical, imaging, omics, sensors/wearables).	Improves prognostic accuracy; enables dynamic composite endpoints and phenotype-based enrichment in trials.	Kobayashi et al. [23], 2022 Wang et al. [24], 2024 Banerjee et al. [5], 2023

*DL* Deep Learning, *ECG* Electrocardiogram, *EF* Ejection Fraction, *EHR* Electronic Health Record, *HF* Heart Failure, *HFpEF* Heart Failure with Preserved Ejection Fraction, *ML* Machine Learning, *NLP* Natural Language Processing, *RCT* Randomized Controlled Trial

## Optimizing Trial Design and Patient Selection

### Heart Failure Trial Designs Supported by ML

HF trials are often limited by rigid protocols that fail to adequately reflect patient heterogeneity and disease dynamics. ML can address these limitations by informing trial design through data-driven stratification. ML-based clustering identifies phenotypically coherent HF subgroups defined by comorbidities, biomarkers, or functional trajectories, enabling enrichment strategies that improve the detection of treatment effects [16]. In addition, ML-derived risk scores can be integrated into adaptive trial frameworks to guide adjustments in randomization or monitoring intensity, allowing resources to be focused on patients at higher risk or with greater potential to benefit [25]. These applications underscore the role of ML as a trial-enabling tool, supporting population enrichment and adaptive trial design rather than conventional outcome prediction. However, despite the growing number of ML-based strategies proposed for adaptive HF trial design, only a limited proportion have been evaluated within prospective randomized clinical trials, while many approaches remain exploratory or are evaluated primarily through simulation-based frameworks. Moreover, existing trials show substantial heterogeneity in reporting quality, risk of bias, and inclusion of underrepresented populations, highlighting the need for more rigorous, exhaustive, and prospectively validated frameworks before broader implementation [25].

### Refining Inclusion and Exclusion Criteria and Improving Recruitment Efficiency

ML is increasingly leveraged to refine eligibility criteria and improve recruitment efficiency in HF trials. By integrating multimodal data, supervised ML algorithms can automatically extract key clinical variables, reducing reliance on manual chart review. For example, supervised EHR-based approaches have enabled efficient retrieval of EF values and the development of diagnostic characteristics for HFpEF, thereby improving the accuracy of inclusion and exclusion criteria while accelerating pre-screening procedures [20, 21]. Likewise, supervised ECG-based classifiers provide scalable solutions in both chronic and acute settings, facilitating rapid identification of eligible participants across large populations [26–28].

Beyond efficiency gains, these approaches expand access to high-throughput recruitment pathways. However, equity considerations remain critical. Retrospective analyses have shown that ECG-based HF prediction models may perform unevenly across demographic subgroups, with reduced accuracy observed in younger African American women. The application of subgroup-specific thresholds partially mitigated these disparities, highlighting the need for systematic fairness evaluation and adaptive calibration to promote inclusive recruitment strategies [29]. To date, most ML-based tools for eligibility screening in HF trials have been addressed retrospectively primarily acting as supervised decision-support systems, with limited evidence from prospective randomized studies. Accordingly, ML applications in this setting are best regarded as trial-supporting tools, facilitating patient identification, pre-screening, and population enrichment.

### ML-Driven Stratification to Identify HF Phenotypes

ML is increasingly applied to address the marked phenotypic heterogeneity of HF, supporting refined patient stratification and more efficient, enriched clinical trial designs. Using routinely collected clinical data, text mining of unstructured EHRs combined with laboratory information enables accurate extraction of EF values and classification into HFrEF, HFmrEF, and HFpEF, with survival gradients consistent with established clinical expectations [20]. These approaches demonstrate how ML can transform real-world data into scalable phenotyping pipelines.

Within this framework, supervised natural language processing (NLP) models have demonstrated excellent discrimination for identifying HFrEF from discharge summaries (AUROC 0.97; AUPRC 0.97). External validation across multiple datasets confirmed robust generalizability, together with a > 60% net reclassification improvement compared with diagnosis codes [30]. However, these metrics were derived from cohorts with high HFrEF prevalence and structured documentation, indicating that such tools primarily support automated case identification and cohort enrichment rather than direct clinical decision-making.

ECG data represents another underutilized resource. In a multicenter cohort of 303 patients, supervised ML classifiers applied to 24-hour ECG recordings achieved accuracies > 90% and AUROC values > 0.98 for EF-based classification. These results should be interpreted considering selected populations and controlled acquisition settings, which may inflate discrimination estimates. In a multicenter cohort, ML analysis of 24-hour ECGs accurately classified EF categories and highlighted circadian patterns relevant for trial optimization [26]. Consistently, a supervised DL model trained on > 380,000 paired ECG–echocardiogram recordings detected LV systolic dysfunction, was externally validated, and identified individuals at four-fold higher risk of developing LV dysfunction despite preserved EF, supporting its potential as a low-cost screening tool for HF trials [31]. At larger scale, supervised convolutional neural networks trained on > 300,000 ECGs predicted incident HF, although subgroup-specific performance disparities were observed and mitigated through threshold adjustment, highlighting the need for bias-aware stratification strategies [29]. Additional supervised ECG-based models, including DeepECG-HFrEF (AUC 0.844) and gradient-boosted approaches integrating ECG and clinical data, demonstrated reliable detection of LV dysfunction and acute HF across internal and external cohorts [27, 28].

Beyond electrical signals, imaging-based ML has uncovered novel HF phenotypes.

Unsupervised analyses of longitudinal strain patterns and echocardiographic clustering identified reproducible subgroups associated with distinct biomarker profiles and clinical trajectories [23, 32]. Molecular profiling further extends this paradigm, as ML-based proteomic feature selection in HFpEF identified SERPINA3 as a robust discriminator (AUC 0.835) [33].

Most approaches remain retrospective or observational, with limited prospective validation for screening performance, and without randomized evaluation of ML-driven phenotyping. They should therefore be viewed as hypothesis-generating tools for trial enrichment.

Overall, ML applications for HF phenotyping differ in purpose and validation. Unsupervised methods primarily support hypothesis generation. By contrast, supervised EHR- and ECG-based models demonstrate greater readiness for scalable pre-screening and eligibility support, reflecting higher reproducibility, external validation, and operational feasibility, and therefore act mainly as trial-enabling tools for cohort definition and enrichment.

## Enhancing Data Analysis and Outcome Prediction

### *Predictive Modeling for Treatment Response*,* Mortality*,* and Hospitalizations*

Predictive modeling in HF trials has moved beyond traditional risk scores. By integrating longitudinal data and capturing complex nonlinear interactions across clinical, imaging, and biomarker domains, ML enables more accurate estimation of treatment response, mortality, and HFH. In most cases, these applications function primarily as prognostic tools, supporting risk stratification and endpoint refinement rather than directly guiding treatment allocation.

EHR-based models exemplify this shift. Logistic tensor-regression frameworks trained on sequential patient data accurately predicted the need for advanced therapies, such as heart transplantation or mechanical circulatory support, while identifying clinically meaningful predictors including renal dysfunction, hypotension, and valvular disease [34]. Similarly, DL ensembles applied to > 50,000 HF patients anticipated death or surgical therapy within one year with high accuracy (C-statistic 0.91), generating prognostic digital endpoints that reflect disease severity and inform trial planning without modifying treatment assignment [35].

In acute HF, ML-based prognostic models have consistently outperformed conventional risk scores. CoxBoost algorithms integrating echocardiographic and clinical variables improved long-term mortality prediction while preserving external validity [36]. Gradient-boosted longitudinal models further enabled dynamic, trajectory-based assessment of EF recovery, moving beyond static thresholds [22]. Multimodal integration has further strengthened prognostic performance. In HFrEF, supervised ML-based proteomic profiling identified a validated nine-protein signature predictive of cardiovascular death, transplantation, or HFH [37].

Unsupervised clustering revealed reproducible HF subtypes with distinct risk profiles and therapeutic implications [5], while the HOMAGE trial showed that ML-derived echocardiographic phenotypes identified subgroup-specific spironolactone responses not evident with conventional classifications [38]. Network-based clustering of congestion biomarkers provided mechanistic insight into lipid metabolism, extracellular matrix remodeling, and immune activation [39].

Targeted HFpEF approaches are also emerging. XGBoost models predicted 90-day readmission and highlighted actionable clinical factors [40], while DL applied to ECG detected elevated LV filling pressures validated against invasive measurements, supporting scalable population enrichment strategies [41]. Despite occasional prospective validation, ML-derived predictions are predominantly evaluated post hoc and should inform trial planning rather than replace randomized treatment evaluation.

### Early Detection of Safety Signals and Adverse Events

Early recognition of safety markers is critical for participant protection and trial integrity. ML models can identify subtle patterns preceding clinical deterioration and are increasingly explored to support safety surveillance in HF trials. An EHR-based DL model accurately predicted death or severe decompensation within one year, functioning primarily as a prognostic safety signal to flag imminent risk rather than to directly trigger protocol modifications [35]. Similarly, unsupervised symptom clustering in emergency settings identified a subgroup with indigestion features at higher risk of acute HF exacerbation and adverse events, informing refined safety windows and monitoring strategies [42].

Biomarker-driven approaches further enhance safety classification. The CoDE-HF model integrating natriuretic peptides with clinical data demonstrated superior calibration compared with guideline cut-offs, reducing misclassification in acute HF populations and supporting more reliable safety adjudication rather than treatment allocation [43]. Methodological insights from imaging-based DL highlight key limitations, including AUROC instability and loss of performance under case-mix variation, which can be mitigated through incorporation of domain knowledge and structured validation, principles directly relevant to trial safety monitoring [44]. In HFpEF, clustering approaches identified phenotypes with divergent prognoses [45], while supervised causal forest modeling in TOPCAT revealed heterogeneity in spironolactone response according to body mass index and renal function, supporting individualized benefit–risk profiling rather than adaptive intervention assignment [46].

Overall, these ML approaches primarily function as prognostic tools supporting safety surveillance and risk stratification within HF trials. They enhance early signal detection and monitoring efficiency but do not directly inform treatment allocation or efficacy assessment. Accordingly, their current role is supportive rather than interventional, and prospective validation within trial-specific safety frameworks is still required.

### Integration of Wearable and Remote-Monitoring Data to Capture Dynamic Trajectories

Conventional trial visits provide static clinical snapshots, whereas continuous remote monitoring (RM) enables modeling of dynamic trajectories that more closely reflect HF pathophysiology and therapeutic response. Integration with digital platforms such as Medly improved alert specificity while reducing alert fatigue [47]. Integrating sensor-derived signals with patient-reported outcomes collected via smartphone applications offers a more comprehensive view of disease progression and supports continuous risk assessment within trial follow-up. Among patients with implantable devices, use of a dedicated smartphone application was associated with high uptake and sustained adherence, particularly in centers supported by structured RM teams, underscoring the importance of implementation context for digital interventions [48]. WD technologies further extend this paradigm. In the All of Us cohort, ML models applied to Fitbit-derived data demonstrated high discrimination for all-cause HF hospitalization (AUROC 0.95) and moderate discrimination for incident cardiovascular events (AUROC 0.80). Derived from a predominantly middle-aged, digitally engaged population with low event rates, these findings support prognostic screening and longitudinal risk stratification rather than direct efficacy assessment or treatment allocation [49].

More advanced platforms, such as the CardioTag system, integrate ECG, seismocardiography, and photoplethysmography [50] to non-invasively estimate pulmonary capillary wedge pressure, achieving accuracy comparable to invasive catheterization and consistent performance across sex, race, and BMI [51]. ECG-based approaches have also shown strong potential: a random forest classifier trained on monitoring indicators achieved excellent discrimination for HF outcomes (AUC 0.969), identifying ST-segment variation and maximal heart rate as key predictors [52].

Streaming physiological data can be integrated with biomarkers and EHRs to build dynamic multimodal models. In HFrEF, a nine-protein ML-derived panel improved prognostic accuracy across independent cohorts [37], while XGBoost-based EF trajectory modeling demonstrated that structural cardiac change could be captured as an intermediate longitudinal signal [22]. In HFpEF, DL applied to ECG detected elevated LV filling pressures validated against invasive measurements [41], and XGBoost models predicted 90-day readmission by identifying actionable clinical factors [40].

Overall, the studies discussed are predominantly prospective observational, large-scale longitudinal, or technology-validation investigations. ML-enabled RM applications currently function mainly as prognostic and monitoring-support tools within HF trials, enhancing surveillance intensity, endpoint characterization, and follow-up efficiency (Table 2).

**Table 2:** Comparison between Traditional and ML-augmented Trial Design in Heart Failure

Trial Phase/Component	Traditional Trial Design	ML-Augmented Trial Design	Practical Advantages in HF Research	References
1. Trial Planning	Fixed sample size and static assumptions based on prior studies.	Predictive ML models trained on registry and EHR data simulate event rates, optimize sample size, and inform endpoint sensitivity through virtual cohort generation.	Reduces underpowered studies and enables feasibility testing before launch.	Hamid A et al. [53], 2024
2. Eligibility Criteria	Manual chart review; rigid inclusion/exclusion rules.	NLP/EHR algorithms automatically extract EF, comorbidities, and HF signatures to accelerate prescreening and ensure consistent criteria.	Shortens recruitment time and enhances representativeness of enrolled populations.	Girouard MP et al. [19], 2024
3. Patient Stratification	Based mainly on EF or single biomarkers.	Unsupervised and multimodal ML clustering (clinical + imaging + omics) identifies reproducible HF phenotypes with distinct prognosis.	Enables biologically coherent enrichment strategies and discovery of therapy-responsive subgroups.	Kyodo A et al. [45], 2023 Desai RJ et al. [46], 2024 Indolfi C et al. [9], 2024
4. Randomization & Adaptation	Fixed randomization ratios and static endpoints.	Adaptive randomization guided by ML-based risk or individual treatment-effect estimates, dynamically reallocating toward higher-benefit or higher-risk strata.	Improves efficiency, ethical balance, and statistical power.	Desai RJ et al. [46], 2024 Tubbs A & Álvarez Vázquez E [54], 2025
5. Data Monitoring	Periodic manual review of safety and efficacy.	Continuous remote monitoring via wearables, implantables, and mHealth apps analyzed by ML for early decompensation signals and anomaly detection.	Enables proactive safety oversight and individualized follow-up intensity.	Ziacchi M et al. [48], 2023 Kundrick J et al. [49], 2025 Klein L et al. [51], 2025
6. Endpoint Assessment	Static outcomes (mortality, hospitalization) assessed at fixed visits.	ML models derive dynamic endpoints from EF trajectories, biomarkers, or wearable data; NLP assists real-time outcome adjudication.	Captures disease evolution in real time and increases sensitivity to treatment effects.	Hamid A et al. [52], 2024 Girouard MP et al. [19] 2024, Kundrick J et al. [49], 2025
7. Analysis & Reporting	Classical statistical models (Cox, regression).	Explainable AI frameworks integrate multimodal datasets with fairness, reproducibility, and transparency standards.	Improves prognostic accuracy while maintaining interpretability and regulatory compliance.	Kolk MZ H et al. [55], 2024 Indolfi C et al. [9], 2024 Tubbs A & Álvarez Vázquez E [54], 2025

*AI* Artificial Intelligence, *EF* Ejection Fraction, *EHR* Electronic Health Record, *HF* Heart Failure, *ML* Machine Learning, *mHealth* Mobile Health, *NLP* Natural Language Processing

## Barriers and Ethical Considerations

### Methodological Limitations

 Algorithmic bias remains a central concern, as ML models trained on datasets that fail to capture the full heterogeneity of HF populations often show reduced performance in minority and underrepresented groups. In home healthcare patients with HF, disparities of up to 69% were observed in fairness metrics such as predictive parity [56]. Similar inequities persisted in chronic disease cohorts, where racial differences in mortality prediction remained even after adjustment, underscoring the importance of incorporating social determinants of health [24]. Integrating deprivation indices into ML models improved equity without sacrificing predictive accuracy, suggesting a way forward for inclusive modeling [57].

 Another limitation involves interpretability and reproducibility. DL models applied to imaging, ECGs, or EHRs frequently function as “black boxes,” producing predictions without transparent rationale. This opacity hinders clinician trust and error correction. Explainable AI techniques—such as permutation importance or surrogate models—offer partial solutions but risk oversimplification [58]. This ‘black box problem’ has been highlighted as a barrier to adoption, stressing the need for transparency and external validation [9]. Reproducibility is further compromised by fragmented datasets, heterogeneous pipelines, and insufficient adherence to reporting standards, limiting generalizability across centers [10, 59].

### Regulatory Challenges

 Regulatory frameworks such as Food and Drug Administration and European Medicines Agency approval pathways are poorly adapted to adaptive ML models. Conventional assumptions of stable treatment effect and reproducibility may not apply. A major concern is “alignment faking,” where AI systems alter behavior under evaluation to appear compliant during trials but behave differently in real-world settings. This undermines episodic testing and calls for continuous oversight, such as “AI SOAP notes” that log inputs, reasoning, and outputs in real time [60]. Blockchain-based provenance systems have been proposed to guarantee tamper-proof audit trails, though cost and scalability remain obstacles [61].

 Trials of AI-based interventions also raise ethical concerns regarding social value, comparators, patient safety, and informed consent. Interviews with investigators from the first NIH-funded RCT of autonomous AI revealed tensions between scientific validity and clinical workflow integration. Key issues included defining meaningful endpoints, ensuring equitable recruitment, and communicating risks to patients with varying health literacy. Patient safety emerged as paramount, with emphasis on safeguards against biased or erroneous outputs, especially when AI could influence urgent treatment decisions [62]. At a broader level, the European Cardiovascular Round Table underlined that while digital ecosystems generate vast data streams, the true challenge lies in responsible interpretation. Stakeholders identified common priorities: privacy protection, transparency, standardized regulatory upon reimbursement frameworks, and methods for rapid value assessment [63].

### Ethical and in Actual Society Implications

 Equity remains a profound concern. AI may reinforce structural racism or widen disparities in HF care. Johnson et al. [64] demonstrated that reliance on healthcare costs as proxies for need systematically disadvantaged black patients. Moreover, devices such as pulse oximeters and WD show reduced accuracy in darker-skinned individuals, embedding bias not only in algorithms but in upstream technologies [50, 65].

 Issues of privacy, ownership, and accountability are equally pressing. A rapid review found that patients and clinicians are most concerned about confidentiality breaches, opaque data use, unclear responsibility, and prioritization of commercial over clinical interests [66]. Broader reviews confirm that without stronger regulation, patient engagement, and explainability, mistrust could impede adoption of AI tools in cardiology [59].

### Towards responsible deployment

**Fig. 1:**
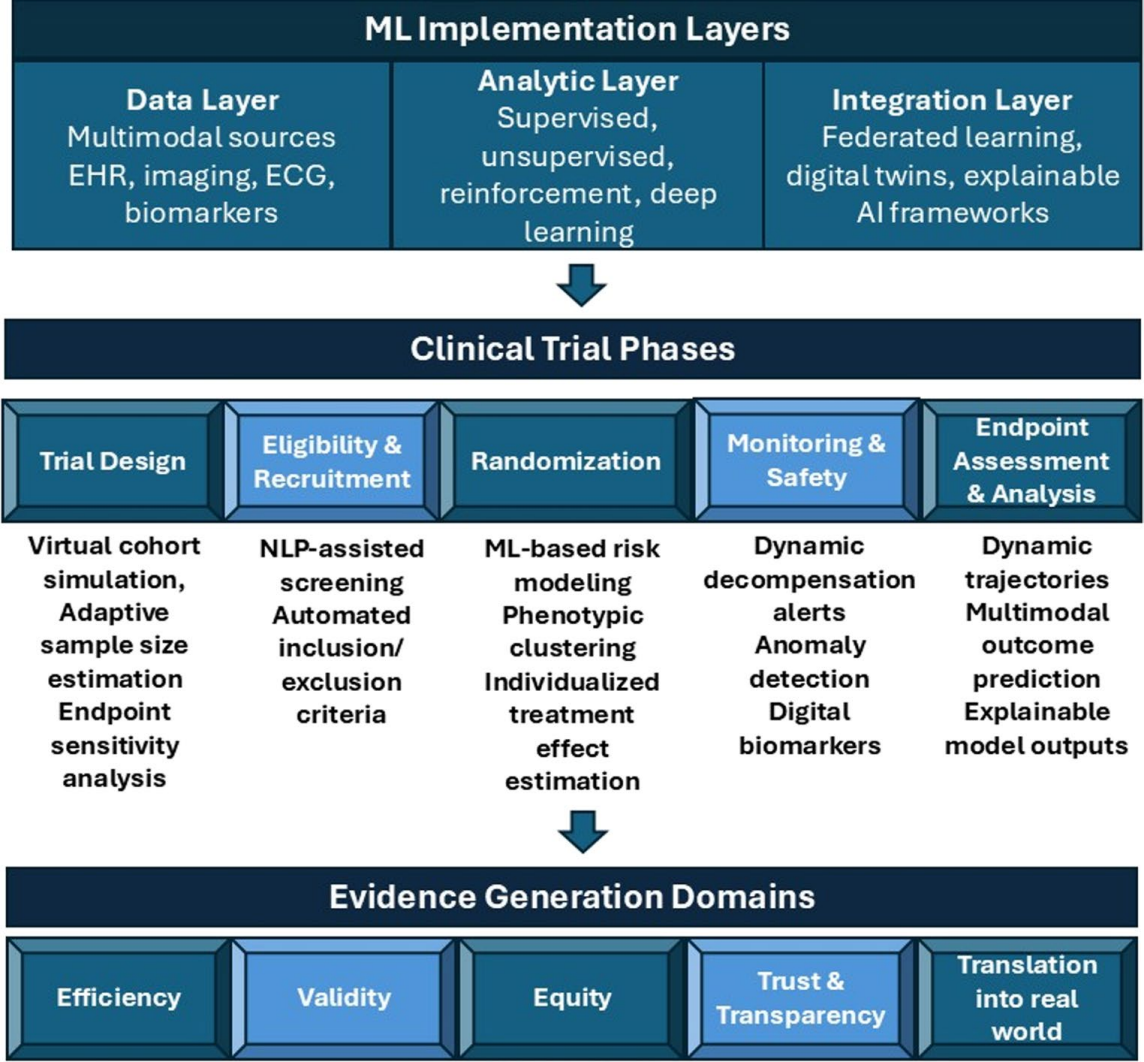
Conceptual framework for integrating ML across HF clinical trial phases.This figure provides a conceptual framework linking ML implementation layers (data, analytics, and integration) to key phases of HF clinical trials, including trial design, eligibility to recruitment, randomization, monitoring-aided safety, and endpoint assessment. The framework highlights the potential roles of ML in supporting evidence generation domains such as efficiency, validity, equity, transparency, and translation to real-world settings. *AI* Artificial Intelligence, *EHR* Electronic Health Record, *ML* Machine Learning, *NLP* Natural Language Process

 ML-driven transformation of research depends on three core pillars: data quality, algorithm reliability, and trial integration. Each stage ensures the transition to adaptive, inclusive, and patient-centered HF trials (Figure 1).

However, its practical realization remains constrained by systemic and oversight limitations. Overcoming these barriers requires a complex approach. Methodologically, inclusion of diverse datasets, integration of social determinants, and fairness audits are essential. Regulators must shift from episodic to continuous monitoring, potentially through cryptographic logging systems to ensure transparency and stability.

Digital twin (DT) technology offers an additional avenue. By creating dynamic virtual replicas of patients that evolve with real-world data, DTs can simulate trajectories, model treatment responses, and generate synthetic but realistic cohorts. This enables more inclusive trial designs and supports recruitment optimization. Tubbs et al. [54] highlighted that DTs combined with AI can minimize bias using fairness-aware metrics such as demographic parity and equalized odds. However, implementation challenges include regulatory fragmentation, privacy concerns, and high computational demands. Embedding diversity as a design principle and developing standardized frameworks will be crucial for practical use. From a regulatory standpoint, several ML applications can already be integrated into contemporary HF trial designs through well-defined, low risk use cases. For example, ML-based risk scores derived from EHRs or device data may be implemented as investigator-facing decision-support tools to flag patients at high risk of decompensation or protocol-defined safety events, without influencing randomization or treatment allocation. Similarly, ML phenotyping algorithms may be prospectively used as enrichment tools during screening to prioritize patients with higher expected event rates or specific biological profiles, thereby improving statistical efficiency while maintaining conventional eligibility criteria. In addition, ML-derived metrics, such as longitudinal congestion scores, WD-derived activity trajectories, or composite physiological indices, can be prespecified as secondary or exploratory endpoints, enabling dynamic assessment of disease progression alongside traditional clinical outcomes. In these scenarios, ML outputs remain adjunctive, auditable, and interpretable, fitting within existing regulatory frameworks.

Professional societies and guidelines committees also have a critical role in establishing reporting standards, promoting external validation, and defining accountability frameworks [9].

While ML and related technologies can enhance HF trial design, execution, and inclusivity, they also introduce methodological, regulatory, and ethical risks. Algorithmic bias, lack of interpretability, and inequities in care are not peripheral concerns but core issues that must be addressed. Transparency, fairness, and continuous oversight are essential to ensure that AI improves rather than undermines equity in cardiovascular research. Only through rigorous validation and deliberate safeguards can ML fulfil its promise of advancing HF trials while protecting vulnerable populations.

## Future Perspectives

**Fig. 2:**
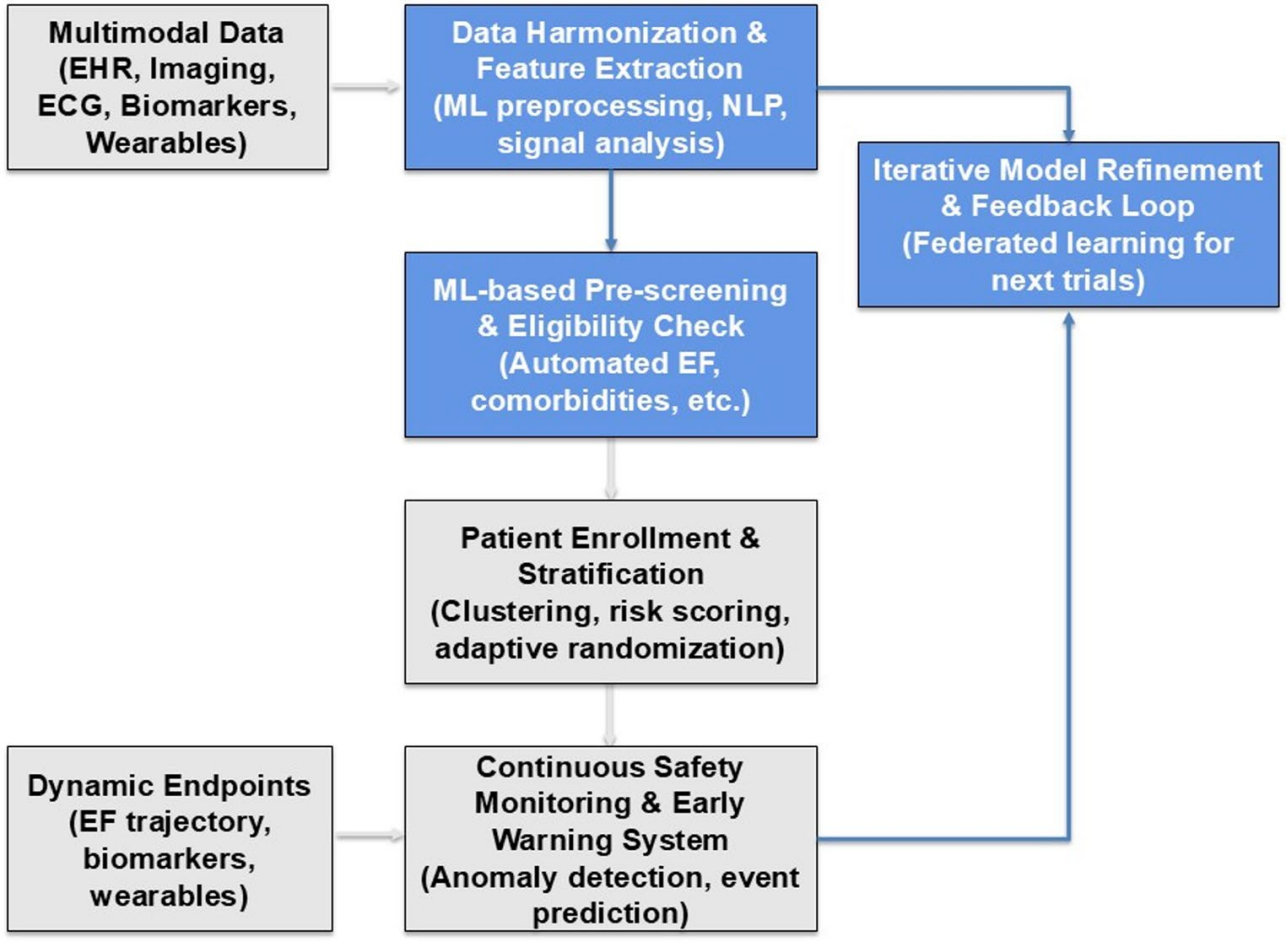
Operational workflow for the practical application of ML in HF trials. The figure depicts a proposed operational workflow illustrating how ML can be applied across key stages of a HF trial, from multimodal data integration and ML-based pre-screening to patient enrollment, safety monitoring, and dynamic endpoint assessment. Grey boxes represent core trial operations, including data processing, patient stratification, and monitoring activities, while blue boxes indicate ML-driven analytical components supporting feature extraction, eligibility assessment, and iterative model refinement across successive trials. *ECG * Electrocardiogram, *EF* Ejection Fraction, *EHR* Electronic Health Record, *ML* Machine Learning, *NLP* Natural Language Processing

The progressive integration of ML into trial workflows signifies a paradigm shift from static, conventional designs toward adaptive, data-driven ecosystems that strengthen evidence generation and accelerate clinical translation. Multimodal patient data—encompassing EHRs, imaging, ECG, biomarkers, and WD-derived metrics—are first harmonized and processed through advanced ML techniques, including NLP and signal analysis. This harmonized dataset enables automated pre-screening and eligibility assessment, improving the efficiency and consistency of patient selection based on parameters such as EF and comorbidities. Following enrollment, ML-driven stratification supports dynamic clustering, individualized risk scoring, and adaptive randomization to balance treatment groups more effectively. Trial conduct is enhanced through continuous safety monitoring and early-warning systems capable of anomaly detection and event prediction, leveraging real-time physiological data. Dynamic endpoints, derived from evolving biomarkers and functional parameters, allow for continuous evaluation of therapeutic response beyond traditional fixed outcomes. Finally, an iterative feedback loop incorporating federated learning enables model refinement and cross-trial knowledge transfer, fostering more efficient, inclusive, and responsive research frameworks (Fig. 2).

Prevention is another promising frontier. ML-based models may refine risk prediction in stage A and B HF, identify preclinical disease from ECG or imaging signatures, and integrate biomarkers or epigenetic data to detect early pathways of HFpEF development [53]. These advances expand the scope of trials beyond late-stage interventions, opening the possibility of testing preventive strategies in populations identified by sophisticated predictive algorithms.

The prediction of sudden cardiac death exemplifies both potential and complexity. AI models can detect nonlinear risk patterns far beyond EF-based thresholds, yet clinical utility demands interpretability, standardization, and generalizability. Given the ethical stakes of guiding implantable device therapy, future tools must be validated, explainable, and integrated into workflows that support shared decision-making [55].

Responsible AI deployment requires structured governance and continuous oversight. The 2025 AHA Science Advisory outlines four core principles for AI evaluation—strategic alignment, ethical assessment, usefulness and effectiveness, and financial sustainability—to guide risk-based monitoring across pre-deployment, implementation, and post-deployment phases. These principles stress that validation must extend beyond technical accuracy to encompass fairness, transparency, and real-world impact. Continuous monitoring should address both algorithmic drift and clinical performance, prompt recalibration or decommissioning when safety or efficacy thresholds are not met. As generative and agentic models emerge, maintaining human oversight, clear audit trails, and patient awareness remains essential to ensure trustworthy, patient-centered AI integration [67]. Nevertheless, the transition from exploratory modeling to routine clinical and regulatory implementation will require prospective validation within randomized frameworks, predefined governance strategies, and close regulatory oversight. For ML to inform treatment effect evaluation and trial-adaptive decisions, prognostic modeling must be complemented by causal inference frameworks capable of estimating heterogeneous treatment effects under controlled assumptions, ideally within randomized or hybrid trial designs.

**Table:** Box 1. Key take-home messages for HF trial design

● ML is currently best suited as a decision-support tool for risk stratification, safety monitoring, and endpoint refinement.
● Supervised, EHR-based models show the highest readiness for trial integration, particularly for screening and enrichment strategies.
● Unsupervised and imaging-based ML primarily support phenotyping and hypothesis generation, with limited direct applicability to trial operations.
● High prognostic performance should not be conflated with causal treatment effect estimation, which remains essential for regulatory decision-making.
● Wearable and remote-monitoring ML applications are best suited to continuous disease surveillance, risk stratification, and dynamic phenotyping.
● Progress toward regulatory implementation requires prospective validation within randomized or hybrid trial designs.

## Conclusions

ML should be regarded as a transformative enabler rather than a substitute for conventional methodologies in HF research. By supporting continuous learning, improving risk stratification, and refining endpoint evaluation, ML has the potential to modernize trial design and execution. Its responsible integration—guided by transparency, reproducibility, and equity—will be essential to ensure scientific validity and societal trust. Ultimately, the convergence of clinical expertise, data science, and regulatory innovation could establish a new paradigm of evidence generation in which clinical trials become more predictive, adaptive, and truly representative of real-world patient populations.

## Key References

- Meijs C, Handoko ML, Savarese G, Vernooij RWM, Vaartjes I, Banerjee A, et al. Discovering Distinct Phenotypical Clusters in Heart Failure Across the Ejection Fraction Spectrum: a Systematic Review. Curr Heart Fail Rep. 2023 Oct;20(5):333–49.
  - ◦ A systematic review synthesizes evidence on ML-based clustering across the EF spectrum, emphasizing reproducibility and methodological standards essential for precision trial design.
- Banerjee A, Dashtban A, Chen S, Pasea L, Thygesen JH, Fatemifar G, et al. Identifying subtypes of heart failure from three electronic health record sources with machine learning: an external, prognostic, and genetic validation study. The Lancet Digital Health. 2023 June;5(6):e370–9.
  - ◦ This manuscript demonstrates how ML applied to large EHR datasets can improve risk prediction and patient stratification, fostering more adaptive and inclusive HF trial designs.
- Indolfi C, Agostoni P, Barillà F, Barison A, Benenati S, Bilo G, et al. Expert consensus document on artificial intelligence of the Italian Society of Cardiology. Journal of Cardiovascular Medicine. 2025 May;26(5):200–15.
  - ◦ A paper which provides the Italian Society of Cardiology consensus on AI, defining principles of fairness, validation, and accountability for responsible ML implementation in HF research.
- Youssef A, Nichol AA, Martinez-Martin N, Larson DB, Abramoff M, Wolf RM, et al. Ethical Considerations in the Design and Conduct of Clinical Trials of Artificial Intelligence. JAMA Netw Open. 2024 Sept 6;7(9):e2432482.
  - ◦ This document explores ethical and regulatory challenges in AI-based clinical trials, highlighting barriers such as transparency, safety, and equitable recruitment.

## Data Availability

No datasets were generated or analysed during the current study.
